# Viromic and Metagenomic Analyses of Commercial Spirulina Fermentations Reveal Remarkable Microbial Diversity

**DOI:** 10.3390/v16071039

**Published:** 2024-06-27

**Authors:** Brian McDonnell, Elvina Parlindungan, Erika Vasiliauskaite, Francesca Bottacini, Keith Coughlan, Lakshmi Priyadarshini Krishnaswami, Tom Sassen, Gabriele Andrea Lugli, Marco Ventura, Felice Mastroleo, Jennifer Mahony, Douwe van Sinderen

**Affiliations:** 1 School of Microbiology, University College Cork, T12 Y337 Cork, Ireland; brian.mcdonnell@ucc.ie (B.M.); erika.vasiliauskaite@ucc.ie (E.V.); keithcoughlan@ucc.ie (K.C.); 119220160@umail.ucc.ie (L.P.K.); j.mahony@ucc.ie (J.M.); 2 APC Microbiome Ireland, University College Cork, T12 YT20 Cork, Ireland; francesca.bottacini@mtu.ie; 3 Biological Sciences, Munster Technological University, Bishopstown, T12 P928 Cork, Ireland; 4 Microbiology Unit, Nuclear Medical Applications, Belgian Nuclear Research Centre, SCK CEN, 2400 Mol, Belgium; felice.mastroleo@sckcen.be; 5 Laboratory of Probiogenomics, Department of Chemistry, Life Sciences, and Environmental Sustainability, University of Parma, 43124 Parma, Italy; gabrieleandrea.lugli@unipr.it (G.A.L.); marco.ventura@unipr.it (M.V.); 6 Interdepartmental Research Centre “Microbiome Research Hub”, University of Parma, 43124 Parma, Italy

**Keywords:** spirulina, virome, metagenome, cyanobacteria, blue-green algae, superfood, *Arthrospira*, *Limnospira*

## Abstract

Commercially produced cyanobacteria preparations sold under the name spirulina are widely consumed, due to their traditional use as a nutrient-rich foodstuff and subsequent marketing as a superfood. Despite their popularity, the microbial composition of ponds used to cultivate these bacteria is understudied. A total of 19 pond samples were obtained from small-scale spirulina farms and subjected to metagenome and/or virome sequencing, and the results were analysed. A remarkable level of prokaryotic and viral diversity was found to be present in the ponds, with *Limnospira* sp. and *Arthrospira* sp. sometimes being notably scarce. A detailed breakdown of prokaryotic and viral components of 15 samples is presented. Twenty putative *Limnospira* sp.-infecting bacteriophage contigs were identified, though no correlation between the performance of these cultures and the presence of phages was found. The high diversity of these samples prevented the identification of clear trends in sample performance over time, between ponds or when comparing successful and failed fermentations.

## 1. Introduction

Spirulina, as we know it, has been consumed traditionally for hundreds if not thousands of years across the globe [1,2]. The first commercially produced food supplement produced under controlled conditions and using the name spirulina was the Linagreen range produced by the DIC corporation in 1978. Since that time, spirulina has been extensively marketed as a superfood, i.e., one that is nutrient rich and generally considered to be beneficial for good health and well-being [3,4,5]. Despite this, the actual content of these products has been a matter of confusion and some debate since their inception, at least in part due to the complexity in cultivating the relevant organisms in the lab environment [2,6]. Spirulina has at different times been referred to as ‘green algae’, ‘blue-green algae’, or as a ‘plant’, but is now known to be a member of the cyanobacteria group. Though it is sold as one product, the actual bacterial content of commercial products may be highly heterogenous, in some cases with over 100 bacterial operational taxonomic units (OTUs) identified [7].

The potential applications of spirulina are diverse, and include agriculture [3,8,9,10,11], aquaculture [12,13,14,15], and human nutrition [5,16], including for those who are immunocompromised (reviewed by [17,18]) and for individuals who live and work in highly pressurised habitats in which space is a major consideration [19]. As a result, spirulina is grown both traditionally (such as by lake surface harvesting and sun drying [20]) and commercially (such as large-scale US and Asian production [21,22]) in many areas worldwide. It is also an important cyanobacterium in numerous ecological niches, and culture breakdown has been implicated in the decline in certain bird populations that rely on it as a food source [23]. Despite this widespread use by humans and generally favourable reputation, concerns regarding its effectiveness [24,25], toxicity [26,27], and contamination with heavy metals persist [4].

A 2019 article [28] shed some much-needed light on the subject of the taxonomic classification of those cyanobacteria that are prepared and sold as spirulina. Among the authors’ findings were that (i) members of the true *Spirulina* genus are not closely related to those species sold under the name, (ii) the genus *Arthrospira* was most cultivated and sold as ‘spirulina’, and (iii) a further genus (*Limnospira*) should be created to encompass commercially grown and sold cyanobacteria. Since that time, additional *Limnospira* genomes have been sequenced and published [29,30], and comparative genomics findings involving members of this genus have been published [31], indicative of expanding interest in this organism. Recent taxonomic undertakings [32,33] provided further clarity on the appropriate nomenclature of this genus and, in line with these, cyanobacterial species grown and sold as spirulina will be referred to as *Limnospira*/*Arthrospira* or *Limnospira platensis* for the purposes of this study.

The biology of bacteriophages infecting *Limnospira* species is an emerging field with only a single lytic phage of the genus described [34] and no complete genome sequences published to our knowledge, though some research on prophages has been performed [35]. Despite this paucity of data, evidence that *Limnospira* defends itself against incoming alien DNA has recently emerged [36], some of which is presumed to be viral in nature, as deduced from the presence of CRISPR-Cas systems and associated CRISPR arrays. Although some of the described CRISPR systems target invading RNA, the majority are known to target double-stranded (ds) DNA. This, combined with the DNA-harbouring nature of the only lytic phage infecting this species described, indicates that *Limnospira-* and *Arthrospira*-infecting phages are likely DNA-harbouring viruses of the *Caudoviricetes* class. Considering the detrimental effect that phages have on the progression and end results of commercial dairy fermentations [37], it is highly likely that phage contamination in commercial spirulina fermentation would have a similar effect. Indeed, bacteriophages of the genus have been proposed as the cause of a breakdown in a major spirulina-driven food chain in a series of African lakes [23], highlighting the importance of this area of study.

The aim of the current study was to analyse the bacterial and viral components of French open ponds used for commercial spirulina cultivation to identify any compositional trends relating to cultivation failure and phage presence. Our work also aimed to elucidate the microbial composition of open spirulina ponds, and, as such, facilitate more detailed safety and effectiveness assessments of cultivations that generate this widely used product.

## 2. Materials and Methods

### 2.1. Sample Processing

Spirulina cultivations from two French farms were carried out in ‘ponds’ approximately 25 m^3^ in volume over a period of one week in a proprietary defined medium consisting (in part) of bicarbonates, phosphates, and nitrates. Individual cultivations were deemed to have failed if growth was observed to have stalled approximately 2–3 days after inoculation (the inoculum consisting typically of a 5 m^3^ sample of a previously successful cultivation). A typical pond sample in the context of this study consisted of 1.5–2 L of liquid medium (containing visible biomass), which was bottled and transported in a cooled container to University College Cork, Ireland (UCC), whereupon it was kept refrigerated (4 °C) until processing. A total of 19 samples (Table 1) were processed individually, 3 of which (S2B1, S2B2, S2B3) had been collected upon cultivation failure.

### 2.2. Metagenome DNA Extraction and Analysis

DNA extraction was performed using a modified version of a commercial kit protocol (Nucleobond AXG 100 with Buffer set III, Macherey-Nagel, Düren, Germany). Presumed cells were firstly pelleted (5000× *g* for 20 min) and pre-treated with lysozyme (final concentration of 0.8 mg/mL; Merck, Darmstadt, Germany) and mutanolysin (final conc. 50 units/mL; Merck) with incubation at 37 °C for one hour. Proteinase K (Macherey-Nagel) was then added to a final conc. of 100 µg/mL, and the samples were incubated at 50 °C for one hour. The remaining protocol was performed as per the manufacturer’s instructions, and DNA was resuspended in 10 mM Tris (Fisher Scientific, Waltham, MA, USA) buffer prior to shipment to the contract sequencing facility.

According to the manufacturer’s instructions, DNA library preparation was performed using the Nextera XT DNA sample preparation kit (Illumina, San Diego, CA, USA). One ng of input DNA from each sample was used for library preparation. The isolated DNA underwent fragmentation, adapter ligation, and amplification. Sequencing was performed by GenProbio, s.r.l. (Parma, Italy) on a NextSeq 550 instrument (Illumina, CA, USA) using a paired-end 150 bp High Output sequencing kit and a deliberate spiking of 1% PhiX control library. Filtered reads were collected and taxonomically classified through the METAnnotatorX2 bioinformatic pipeline [38] using the up-to-date genome RefSeq database retrieved from NCBI (www.ncbi.nlm.nih.gov). DNA sequences were subjected to whole-metagenome assembly using Spades v3.14 [39] with default parameters and the metagenomic flag option (--meta) together with k-mer sizes of 21, 33, 55, and 77. METAnnotatorX2 [38] classified, at the species level, those reads with a nucleotide identity of >94% to reference genomes, normalising species abundances based on the reference genome size.

### 2.3. Virome DNA Extraction and Analysis

Virome DNA extraction was performed using a method established by UCC (adapted from [40,41],) by firstly enriching for viral particles followed by DNA extraction. Firstly, 200 mL of each sample was centrifuged at 5000× *g* for one hour. The supernatant was then treated with NaCl (Merck, Germany; to 1 M) for one hour at 4 °C on a rotary shaker. Samples were then centrifuged at 28,000× *g* for 15 min or 10,000× *g* for 35 min, followed by double filtration (firstly using 0.45 µm pore size filters, followed by 0.2 µm). Viral particles were precipitated with PEG8000 (Merck) at a final concentration of 10% on a rotary shaker overnight at 4 °C. Following precipitation, the samples were centrifuged at 10,000× *g* for 25 min and the pellet was resuspended in 1 mL of SM buffer [42]. DNase treatment (20 units/mL) was performed at room temperature for 15 min to remove any remaining contaminating host DNA. The DNAse was then inactivated at 75 °C for 10 min. Viral DNA extraction was then performed using a Norgen phage DNA extraction kit (Norgen Biotek Corp., Thorold, ON, Canada), as per the manufacturer’s instructions.

Library preparation and sequencing was performed according to the metagenome analysis protocol described above. Filtered reads were collected and taxonomically classified through the METAnnotatorX2 [38] pipeline using the up-to-date genome RefSeq and Virus RefSeq databases retrieved from NCBI and assembled as described above. 

All assembled contigs were then submitted to the PhaBOX online server [43], an integrated web server which incorporates phage contig identification by PhaMer [43], taxonomy classification by PhaGCN [44], host prediction using Cherry [45], and lifestyle prediction by PhaTYP [46]. Standard PhaBOX parameters were used for all analyses. The relative abundance of individual viral sequences was determined by establishing the Reads Per Kilobase per Million mapped reads (RPKM) of each contig using CoverM version 0.4.0 (B. Woodcroft, unpublished, https://github.com/wwood/CoverM) contig RPKM method, with minimum read % identity, minimum read aligned %, and minimum covered fraction all set to 80%. The manual curation of PhaBOX and CoverM outputs enabled the assessment of overall phage diversity, individual phage contigs, and trends in viral presence/absence and abundance across multiple ponds and time points.

## 3. Results and Discussion

### 3.1. Metagenome Sequencing

**Figure 1:**
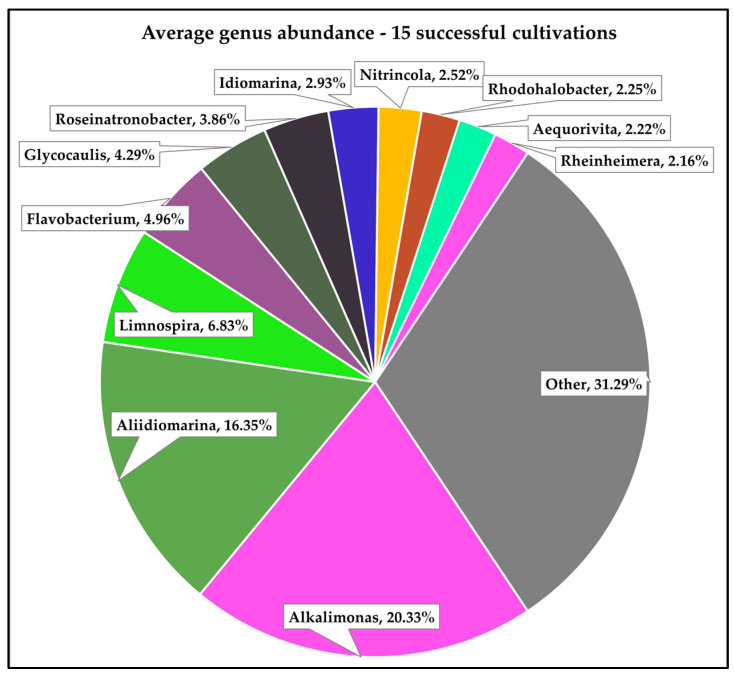
% relative abundance (normalised) of bacterial genera across 15 distinct successful spirulina cultivations.

The metagenomic analysis of a total of 15 pond samples (all successful cultivations) resulted in between 5815 (sample S2B3-21-9) and 18,632 (sample S2B3-7-1-22) classified reads (Table 1). Those reads classified as bacteria were further subclassified (as per Section 2) into 90 distinct bacterial genera and sorted by % relative abundance per sample, corrected for genome size. As expected, no reads resulting from the metagenomic sequencing were classified as viral. A snapshot of the distribution of the bacterial component of these reads across all 15 samples is provided in Figure 1, with the number and proportion of those reads which were assigned to either the *Limnospira* genus or the *Limnospira indica* species given in Table 1. Interestingly, the number of reads assigned to the *Limnospira* genus were generally in the minority, comprising 7% of the total number of reads classified as bacteria (% per sample given in Table 1). This result was unexpected given that *Limnospira* is the bacterial genus intended to be cultivated. This may indicate (i) a limitation of the DNA extraction method employed and/or (ii) a higher level of diversity in these cultivations than might have been predicted prior to analysis. 

Aside from *Limnospira*, the most abundant bacterial genera detected were *Alkalimonas* (20% of total bacterial reads), *Aliidiomarina* (16%), and *Flavobacterium* (5%), with *Glycocaulis* and *Roseinatronobacter* comprising 4% of reads each (Figure 1). *Alkalimonas* and *Aliidiomarina* species are halophilic and alkaliphilic, though they may be found in a range of environments [47,48] such as alkaline soil [49,50], soda lakes [51,52], and the deep sea [53,54]. Flavobacteria are most widely known as fish pathogens [55] and have consequently been found to inhabit freshwater, saltwater, and ice [56]. Interestingly, species in the *Glycocaulis* genus have been described in samples originating from hydrothermal vents [57] and other extreme environments such as crude oil [58] and the Mariana Trench [59], while Roseinatronobacteria are commonly found in soda lakes [60,61] and reportedly in aquatic spring environments of up to pH 12 [62]. Considering that the ponds analysed in this study are not exposed to environments such as these, it is reasonable to assume that the presence of a wide variety of bacterial genera therein is attributable to co-inoculation with *Limnospira* spp., which are found to naturally inhabit similar environments [31,33,63]. The identification of this highly diverse cohort of Gram-negative bacteria, though not entirely expected, is reminiscent of previous metagenomic analyses of commercial spirulina products [7]. 

### 3.2. Virome Sequencing

The study of the virome of a particular environment has been employed in various studies as a method to ascertain its microbial composition, diversity, and population dynamics [64,65,66,67,68]. As such, a virome study was undertaken on the 19 pond samples (Table 1) in the present study to elucidate their viral and putative host composition. As dictated by the method employed, the scope of this analysis was limited to DNA-harbouring bacteriophages.

Virome reads were generated and assembled as per Section 2, producing a total of 6500 individual contigs. Following PhaBOX (and, in particular, PhaMer) analysis, these were further filtered into phage and non-phage contigs. Interestingly, the majority (65% or 4241) of contigs were designated as non-phage by PhaMer, despite efforts to remove as much host/bacterial DNA as possible from the preparation (see Section 2). Separate taxonomic profiling of the virome reads indicated that, similarly to the metagenomic analysis, sequences associated with *Limnospira*, *Glycocaulis*, *Flavobacterium,* and *Idiomarina* (amongst other genera) were detected; however, it is not known whether these reads corresponded to the ‘non-viral’ cohort or if these corresponded to integrated prophages. This finding was not necessarily surprising given the relative amounts of bacterial and viral DNA in a given environmental sample, and it is a known issue in virome studies [69]. In this sense, PhaMer proved to be an exceedingly useful tool which identified and segregated 2259 phage contigs rapidly as part of the PhaBOX toolset. 

**Figure 2:**
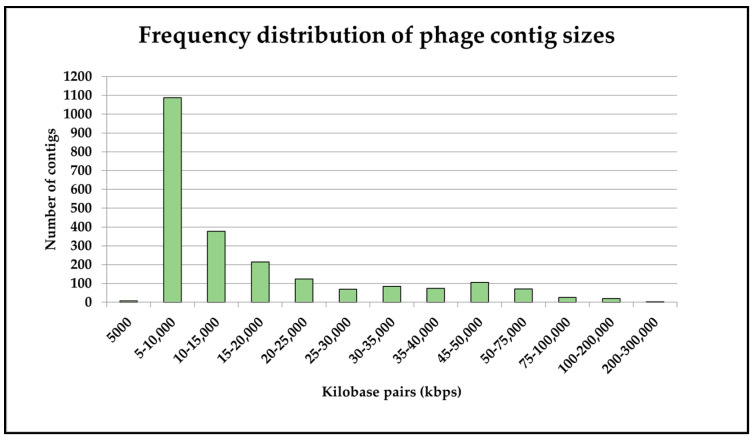
Frequency distribution of viral contig sizes generated using the described sequencing and assembly methods.

Following this assignment, we conducted an analysis on the spread of contig sizes in the above set, whereupon it was found that the majority of viral contigs identified were between 5000 and 10,000 bp in length (Figure 2). A small number (n = 3) of viral contigs were over 200,000 bp in length. Two of these were assigned to the newly created *Kyanoviridae* family and one to the newly created *Straboviridae* family [70], both of which incorporate T4-like phages which were previously classed as *Myoviridae*. The two *Kyanoviridae* phages were predicted (by CHERRY) to infect members of the *Flavobacterium* genus, though the majority of this phage family are known to infect *Synechococcus*, based on a manual search of the NCBI Virus database (https://www.ncbi.nlm.nih.gov/labs/virus/vssi/#/, accessed on 1 March 2024). The third (*Straboviridae*) phage was predicted to infect *Streptococcus cristatus*, a human oral bacterium [71], for which no lytic phages have yet been described to our knowledge.

**Figure 3:**
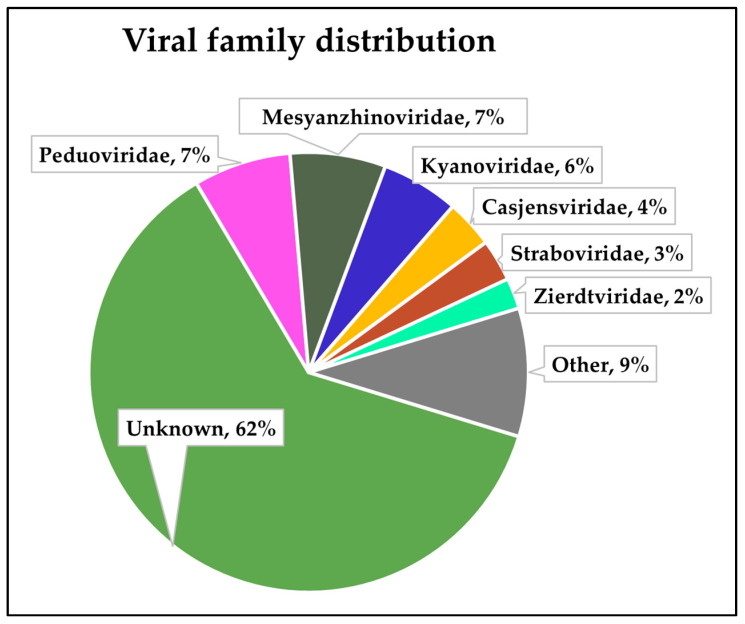
Proportion of viral families amongst assembled phage contigs.

These 2259 identified viral contigs were then classified into known viral families, the distribution of which is presented in Figure 3. A large proportion of the contigs (62%) were not classified, and a visual assessment of the distribution suggested that those ‘unknown’ family contigs fell largely in the 5000–10,000 kbp size group, likely limiting the ability of PhaGCN to assign families correctly [44].

**Figure 4:**
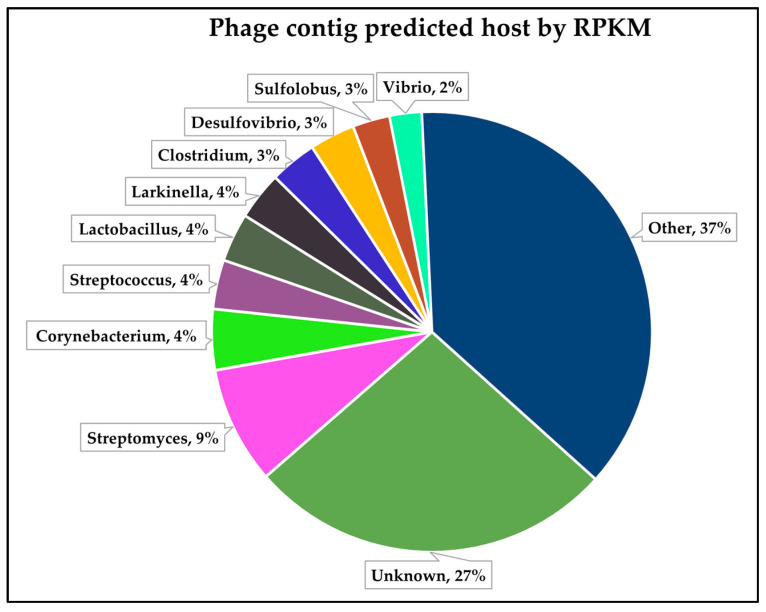
Distribution of predicted phage hosts by relative abundance (RPKM).

Phage host assignment has long been a challenge in the field of metagenomics, and a number of tools have emerged in the last decade to try and overcome this serious research bottleneck [72,73,74,75,76]. Of these, PhaBOX employs CHERRY [45], which claims to have an accuracy of 80% and also to outperform other currently available computational models. Figure 4 shows the proportion of host genera assigned to individual phage contigs in descending order by relative abundance (as based on a RPKM assessment), determined as per Section 2. Evident is the absence of conformity between those genera predicted by metagenomic analysis and those predicted by phage host assignment across the overall sample set. This disparity may have multiple explanations: (i) a potential bias towards Gram-negative bacteria in the metagenomic analysis, as discussed above; (ii) the large proportion (27%) of contigs for which CHERRY could not assign a host; or (iii) the enormous diversity in hosts assigned by CHERRY, i.e., the ‘other’ group (37%) in Figure 4, which represents a total of 468 distinct bacterial genera assigned as phage hosts. Despite these potential limitations, CHERRY analysis may be useful in developing an overall snapshot of the viral diversity in a given sample, when used in combination with PhaMer and PhaGCN.

### 3.3. Identification and Analysis of Putative Limnospira-Infecting Phages

To date, only a single *Limnospira*-infecting phage has been characterised [34] and neither the genome of this phage, nor of any other, has been sequenced. In total, across all samples, 20 contigs were assigned to either *Limnospira indica*, *Limnospira maxima*, or *Arthrospira platensis* as putative hosts by CHERRY, the characteristics of which are presented in Table 2. Manual searches using BLASTn (https://blast.ncbi.nlm.nih.gov/, accessed on 1 February 2024) allowed similar contigs to be identified, and those sharing 97% nucleotide identity over at least 50% of the contig have been colour-coded accordingly in Table 2 to give a broad indication of the diversity of these putative phages.

With the aforementioned limitations of phage host prediction tools in mind, each contig was subjected to a manual blastn [77] analysis in an attempt to verify the host prediction performed by CHERRY. In the majority of cases, the contigs were most similar at the nucleotide level to members of the *Limnospira* and (former) *Arthrospira* genus.

**Figure 5:**
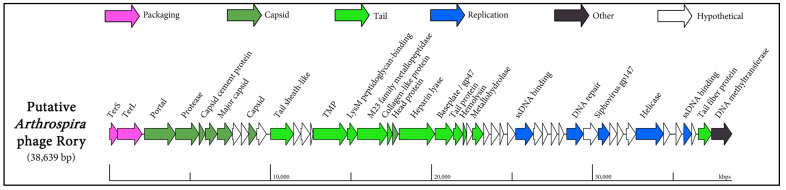
Schematic diagram of the viral metagenome assembled genome (MAG) of putative *Arthrospira platensis* Rory phage.

One contig which did not exhibit significant similarity to any sequence in the NCBI database was Node_28, a predicted virulent phage of *Arthrospira platensis*. This putative phage was subjected to further scrutiny due to its size, with 38.6 kb being of sufficient length to harbour the major functional modules usually found in phage genomes, such as those encoding the necessary proteins for DNA replication, DNA packaging, and virion assembly (a schematic diagram of this phage (termed Rory) genome is provided in Figure 5).

Considering that no genome of a virulent phage of *Arthrospira*/*Limnospira* has been described thus far, we endeavoured to confirm the host specificity assigned by CHERRY. To this end, the entire nucleotide contig was supplied to three further and distinct web-based phage host prediction tools, the results of which are given in Table 3.

It is evident from the results presented in Table 3 that we were unable to conclusively assign a bacterial host to this phage, given that the four different tools tested not only assigned different bacterial species as the potential host, but different genera, families, orders, classes, and phyla. These results align with the enormous effort that has been placed into developing bioinformatic tools to solve this problem. Further studies in this area should aim to reconcile the variety of bioinformatic methods used to assign bacterial hosts to unknown phages, but in the case of *Limnospira* phages, this will likely also require traditional microbiological techniques to establish a definitive infection profile.

## 4. Conclusions

Artisanal foods are coming under increasing scrutiny with regard to microbiological diversity as well as from a safety perspective [82,83,84,85,86,87]. In the present study, we investigated the metagenomes and viromes of a number of open ponds which are used to grow spirulina on a relatively small scale. As such, virome sequencing of 19 pond samples and metagenomic sequencing of 15 pond samples was performed and the results analysed. A large amount of diversity across the prokaryotic and viral content of the ponds was exhibited by most of the samples provided, with *Limnospira*/*Arthrospira* genera apparently in the minority. Several distinct genera were found to be present that were postulated to have originated from those environments in which *Limnospira* spp. can also be found, i.e., in the initial pond inocula (the source of which is currently unknown). Further studies around spirulina cultivation will be useful in establishing if this bacterial profile is maintained in other spirulina farms, which in turn may shed further light on the relationships, if any, between these genera.

Twenty putative *Arthrospira*- or *Limnospira*-infecting phage contigs were identified and analysed, with a single contig being subjected to detailed analysis including gene prediction and annotation because of its large size. The area of host assignment to unknown putative phages continues to pose difficulties. Host assignment of this putative phage was attempted using various online tools as well as manual curation but could not be resolved unambiguously. Further studies in this area will likely require the isolation of *Limnospira*-infecting phages in the laboratory environment to conclusively verify their infectivity profiles, prior to phenotypic and genotypic analyses, greatly expanding the current knowledge base. The requirement for scientifically informed strategies for the mitigation of phage-induced spoilage will undoubtedly increase concomitantly with the popularity of spirulina and other culture-based foodstuffs.

## Figures and Tables

**Table 1:** Sample information and absolute filtered read numbers generated by metagenome and virome sequencing of pond samples. Reads taxonomically assigned to the *Limnospira* genus are given in absolute numbers and as a percentage of overall metagenomic reads, providing an indication of their abundance in each sample. ‘-’; not calculated. ^1^ = collected from Farm 1 (all other samples collected from Farm 2).

Sample Name	Date of Sampling	Sequenced Reads	Filtered Reads	Classified Reads		*Limnospira* spp. Reads (Metagenome)	as % of Total Metagenome Reads
2b ^1^	August 2018	56,581	39,990	Virome	7842	-	-
S2B1 (failure)	September 2021	191,356	75,833	Virome	7453		
S2B2 (failure)	September 2021	5,523,647	4,432,696	Virome	3575		
S2B3 (failure)	September 2021	3,018,468	2,809,813	Virome	3233		
S1B2-21-9	September 2021	29,963	28,342	Metagenome	14,871	0	0.00
		5,877,945	5,716,372	Virome	5292	-	-
S1B2-oct	October 2021	25,520	24,044	Metagenome	14,671	3006	20.49
		6,021,430	5,862,093	Virome	5970	-	-
S1B2-1-22	January 2022	22,502	21,011	Metagenome	10,981	641	5.84
		3,894,117	3,814,643	Virome	3388	-	-
S1B7-21-9	September 2021	25,548	24,186	Metagenome	10,527	0	0.00
		5,924,463	5,747,853	Virome	14,079	-	-
S1B7-1-22	January 2022	16,765	15,244	Metagenome	8291	335	4.04
		4,232,472	4,123,599	Virome	2936	-	-
S2B14-21-9	September 2021	27,112	25,573	Metagenome	14,466	0	0.00
		4,151,790	4,079,681	Virome	2740	-	-
S2B14-11-21	November 2021	22,998	20,743	Metagenome	10,880	300	2.76
		2,582,342	2,520,442	Virome	1392	-	-
S2B14-Dec-21	December 2021	18,847	17,491	Metagenome	9978	304	3.05
		4,741,802	4,628,992	Virome	1744	-	-
S2B3-21-9	September 2021	15,712	13,022	Metagenome	5815	0	0.00
		4,064,953	3,983,395	Virome	2310	-	-
S2B3-oct	October 2021	20,305	18,817	Metagenome	8911	1451	16.28
		4,680,796	4,455,834	Virome	20,406	-	-
S2B3-7-1-22	January 2022	31,805	30,038	Metagenome	18,632	4477	24.03
		4,141,294	4,035,443	Virome	6323	-	-
S2B7-oct	October 2021	19,848	17,262	Metagenome	6650	573	8.62
		5,461,758	5,294,359	Virome	5147	-	-
S2C1-21-9	September 2021	23,440	22,129	Metagenome	13,325	999	7.50
		4,023,482	3,921,215	Virome	7782	-	-
S2C1-oct	October 2021	23,308	20,753	Metagenome	8174	119	1.45
		3,732,816	3,473,164	Virome	6965	-	-
S2C1-1-22	January 2022	17,192	15,584	Metagenome	6930	983	14.18
		2,131,399	2,090,652	Virome	2461	-	-

**Table 2:** Contiguous sequence (contig) analysis of virome data. Contigs exhibiting amino acid identity to bacteriophage sequences are listed, along with the sizes of these contigs and the samples from which they were derived. Contigs exhibiting > 97% nucleotide identity to each other are coloured similarly and in adjacent rows for comparative purposes.

Sample	Accession	Length	PhaMer	PhaTYP	PhaGCN	CHERRY	Top Hit (Blastn)
2b	NODE_36_length_16638_cov_4.583057	16,638	phage	virulent	unknown	*Limnospira indica*	No hit
S1B2-21-9	NODE_112_length_13274_cov_6.270061	13,274	phage	virulent	unknown	*Arthrospira platensis*	Uncultured *Caudovirales* phage
S1B2-oct	NODE_531_length_5912_cov_158.66289	5912	phage	virulent	unknown	*Arthrospira platensis*	No hit
S1B7-1-22	NODE_323_length_5912_cov_106.02519	5912	phage	virulent	unknown	*Arthrospira platensis*	No hit
s2b3-7-1-22	NODE_396_length_8718_cov_28.387802	8718	phage	virulent	unknown	*Arthrospira platensis*	No hit
s1b7-21-9	NODE_370_length_7665_cov_20.110174	7665	phage	virulent	unknown	*Limnospira maxima*	*Arthrospira platensis* C1 chromosome
S1B2-21-9	NODE_240_length_5397_cov_2.393985	5397	phage	virulent	unknown	*Limnospira maxima*	*Arthrospira platensis* C1 chromosome
s2b3-oct	NODE_485_length_7782_cov_19.334848	7782	phage	virulent	unknown	*Limnospira maxima*	*Arthrospira platensis* C1 chromosome
s2b7-oct	NODE_238_length_7533_cov_3.470494	7533	phage	virulent	unknown	*Limnospira maxima*	*Arthrospira platensis* C1 chromosome
S1B2-oct	NODE_439_length_7299_cov_6.697591	7299	phage	temperate	unknown	*Limnospira maxima*	*Arthrospira* sp. TJSD092 chromosome
s1b7-21-9	NODE_382_length_7496_cov_22.413668	7496	phage	virulent	unknown	*Limnospira maxima*	*Arthrospira platensis* C1 chromosome
s2b3-oct	NODE_517_length_7292_cov_23.274012	7292	phage	temperate	unknown	*Limnospira maxima*	*Arthrospira platensis* C1 chromosome
s2b3-7-1-22	NODE_469_length_7495_cov_5.71420	7495	phage	virulent	unknown	*Limnospira maxima*	*Arthrospira* sp. TJSD092 chromosome
s1b7-21-9	NODE_261_length_11129_cov_16.54904	11,129	phage	temperate	unknown	*Arthrospira platensis*	*Limnospira fusiformis* SAG 85.79 chromosome
s2b3-oct	NODE_379_length_10119_cov_16.70065	10,119	phage	temperate	unknown	*Arthrospira platensis*	*Limnospira fusiformis* SAG 85.79 chromosome
s1b7-21-9	NODE_461_length_6172_cov_15.65381	6172	phage	virulent	unknown	*Limnospira maxima*	*Arthrospira* sp. TJSD092 chromosome
s2b3-7-1-22	NODE_281_length_11272_cov_4.703082	11,272	phage	virulent	unknown	*Limnospira maxima*	*Limnospira fusiformis* KN01
s2b3-oct	NODE_321_length_12009_cov_19.52816	12,009	phage	temperate	unknown	*Limnospira maxima*	*Arthrospira platensis* C1 chromosome
s2b3-oct	NODE_463_length_8258_cov_20.730962	8258	phage	temperate	unknown	*Limnospira maxima*	*Arthrospira* sp. TJSD092 chromosome
s2b14-21-9	NODE_28_length_38639_cov_12.619237	38,639	phage	virulent	unknown	*Arthrospira platensis*	No hit

**Table 3:** Host assignment of Rory phage by four distinct web server prediction tools.

Web Server	Host Prediction Tool	Host Assignment
PhaBOX [43]	CHERRY [45]	*Arthrospira platensis*
PhageScope [78]	DeepHost [73]	*Salmonella enterica*
PhaGAA [79]	vHULK [80]	*Escherichia coli*
PhageTB [81]	Custom; BLAST [77]	*Mycolicibacterium smegmatis*

## Data Availability

Raw virome and metagenome reads, as well as assembled virome contigs, have been uploaded to the Sequencing Reads Archive (SRA) at NCBI and are available under BioProject number PRJNA1114024.
